# Haplotype analysis of common variants in the *BRCA1 *gene and risk of sporadic breast cancer

**DOI:** 10.1186/bcr973

**Published:** 2004-12-16

**Authors:** David G Cox, Peter Kraft, Susan E Hankinson, David J Hunter

**Affiliations:** 1 Department of Epidemiology, Harvard School of Public Health, Boston, Massachusetts, USA; 2 Program in Molecular and Genetic Epidemiology, Harvard School of Public Health, Boston, Massachusetts, USA; 3 Channing Laboratory, Department of Medicine, Brigham and Women's Hospital and Harvard Medical School, Boston, Massachusetts, USA

**Keywords:** breast cancer, *BRCA1*, haplotype, single nucleotide polymorphism

## Abstract

**Introduction:**

Truncation mutations in the *BRCA1 *gene cause a substantial increase in risk of breast cancer. However, these mutations are rare in the general population and account for little of the overall incidence of sporadic breast cancer.

**Method:**

We used whole-gene resequencing data to select haplotype tagging single nucleotide polymorphisms, and examined the association between common haplotypes of *BRCA1 *and breast cancer in a nested case-control study in the Nurses' Health Study (1323 cases and 1910 controls).

**Results:**

One haplotype was associated with a slight increase in risk (odds ratio 1.18, 95% confidence interval 1.02–1.37). A significant interaction (*P *= 0.05) was seen between this haplotype, positive family history of breast cancer, and breast cancer risk. Although not statistically significant, similar interactions were observed with age at diagnosis and with menopausal status at diagnosis; risk tended to be higher among younger, pre-menopausal women.

**Conclusions:**

We have described a haplotype in the *BRCA1 *gene that was associated with an approximately 20% increase in risk of sporadic breast cancer in the general population. However, the functional variant(s) responsible for the association are unclear.

## Introduction

Truncation mutations in the *BRCA1 *gene are high-penetrance, low-prevalence factors in risk of breast cancer. *BRCA1 *is hypothesized to be a locus under recombinational inhibition, and very few haplotypes have been described. In fact, only one haplotype block and two major haplotypes have been shown to exist in Caucasians. Because of the size of the gene (more than 80 kilobases), polymorphism discovery screenings have focused on exons. Although many non-synonymous polymorphisms are known in the gene, the degree of linkage disequilibrium (LD) across the entire region limits genetic variability. This limited variability has led to inconclusive results in the risk of sporadic breast cancer associated with variants in the *BRCA1 *gene [1,2].

The high degree of LD at *BRCA1 *led Huttley and colleagues [3] to investigate the possibility of recent selective pressure being exerted on this gene. They found that whereas the ratio of non-synonymous to synonymous nucleotide substitutions is the same between the chimpanzee and humans, this ratio is different from other primates, and greater than 1. They also note that these differences occur in the region of *BRCA1 *that interacts with Rad51, suggesting that it is the role of BRCA1 in maintaining genome integrity that has driven this selection.

Paradoxically, *BRCA1 *has a large number of Alu repeat sequences. These are repetitive elements that are thought to be involved in recombination and evolution of the genome [4,5]. Given that knocking out *brca1 *in mice is embryonic lethal [6], it can be hypothesized that the apparent suppression of recombination at *BRCA1 *in the human is due to the non-viability of recombinants.

Recently, resequencing information over the entire region of the gene, including most introns, has become publicly available [7]. We used these data to select haplotype tagging single nucleotide polymorphisms (htSNPs), to test the association of these haplotypes with breast cancer risk in a nested case-control study within the Nurses' Health Study.

## Method

Resequencing information from the Environmental Genome Project of the National Institute of Environmental Health Sciences (NIEHS) at the University of Washington was used to generate haplotypes for the selection of htSNPs [7]. There were 90 individuals with 301 SNPs in the whole data set. SNPs were excluded from analysis if they were out of Hardy–Weinberg equilibrium (*P *< 0.05), had a minor allele frequency of less than 5%, or had more than 25% missing data. Haplotypes were reconstructed with PHASE [8], and htSNPs were determined with BEST [9]. Four htSNPs were selected, at positions 33,420 (rs799917, P871L), 38,085 (rs8176166), 44,059 (rs3737559), and 64,646 (rs8176267, base pairs reported as on GenBank sequence AY273801).

These htSNPs were genotyped in cases and controls using the TaqMan system (Applied Biosystems, Foster City, CA). Primer and probe sequences are available from the authors on request. Our study consisted of 1323 breast cancer cases and 1910 controls, nested within the prospective Nurses' Health Study. The Nurses' Health study was initiated in 1976, when 121,700 United States registered nurses between the ages of 30 and 55 years returned an initial questionnaire reporting medical histories and baseline health-related exposures. Updated information has been obtained by questionnaire every 2 years. Incident breast cancers were identified by self-report and confirmed by medical record review. Between 1989 and 1990, blood samples were collected from 32,826 women. Follow-up has been about 98% in all subsequent questionnaire cycles for this subcohort. Eligible cases in this study consisted of women with incident breast cancer from the subcohort who gave a blood specimen. Cases with a diagnosis any time after blood collection up to 1 June 2000 with no previously diagnosed cancer except for nonmelanoma skin cancer were included. Controls were randomly selected participants who gave a blood sample and were free of diagnosed cancer (except nonmelanoma skin cancer), and were matched to cases on the basis of age, menopausal status, recent post-menopausal hormone use, and time, day, and month of blood collection. Table 1 shows basic characteristics of cases and controls.

Haplotype frequencies were estimated with the EM algorithm, as implemented in SAS PROC Haplotype (SAS Institute, Cary, NC). Omnibus tests of haplotype association and haplotype-specific odds ratios (ORs) were calculated by haplotype replacement regression [10], assuming an additive model using the probability of carrying each pair of haplotypes provided by PROC Haplotype. The most common haplotype was used as the reference, and rare haplotypes (combined frequency less than 0.5%) were dropped from analysis. Unconditional logistic regression analyses were used to determine relative risk, controlling for age, family history of breast cancer, history of benign breast disease, post-menopausal hormone use, parity, age at first birth, and age of menopause. We assumed an additive model, where haplotype-specific parameters represent the per-haplotype increase in log odds of disease. Departures from a multiplicative gene × environment interaction model were tested by means of likelihood ratio tests.

A fifth SNP (Q356R, rs1799950), previously described as being associated with a reduced risk of breast cancer [1], was also examined with a TaqMan assay. This SNP was not present at more than 5% in the resequencing data and therefore was not included in our haplotype analysis. All *P *values reported are two sided.

Sequence alignments were performed with base pairs 64,601–64,700 on GenBank sequence AY273801. Blast alignments were performed over the web at  using the blastn program against the alu_repeats database. No filtering was used, and expected values were set at 10^-20 ^to limit the number of hits. All other default values were used. AluSp repeats on contig NT_010755 were selected from the AluGene database . These sequences were aligned with ClustalW at , using all default values.

## Results and Discussion

The polymorphism at codon 356 in the *BRCA1 *gene had previously been described as being inversely associated with breast cancer risk (Gln356→ Arg, OR 0.88, 95% confidence interval [CI] 0.63–1.23; Arg356→ Arg, OR 0.00, 95% CI 0.00–0.56) [1]. We were unable to reproduce these results in our data set. Dunning and colleagues did not observe any homozygotes of the Arg allele at this codon among cases (*n *= 765). In contrast, we observed homozygotes among both cases and controls, and did not detect any association (Table 2). We had about 80% power to detect a relative risk of 0.73 assuming a log additive model. This polymorphism was not detected above the 5% threshold for inclusion as a htSNP in the NIEHS database, and was not included in our haplotype analyses. We did explore its inclusion in the haplotype analyses, and it did not materially alter the risk estimates for other haplotypes.

Five haplotypes of more than 5% frequency were described from the 39 polymorphisms meeting the selection criteria. *BRCA1 *exists as one haplotype block, with significant LD along the entire gene. Only four SNPs were needed to tag these haplotypes. To test the hypothesis that a difference in haplotype frequencies is seen between cases and controls, a global test was performed (*P *= 0.08). This test is not formally significant; this should be kept in mind while interpreting results based on haplotype analysis. Table 3 shows the results of the regression trend test of haplotypes.

Haplotype 2 (C A G G) was associated with a small, though significant, increase in risk (OR 1.18, 95% CI 1.02–1.37; Table 3). When considering the diplotype of haplotype 2, a significant increase in risk was observed among the homozygous carriers (OR 1.62, 95% CI 1.05–2.48; Table 4). A nearly significant interaction was seen between haplotype 2 and family history of breast cancer (*P *= 0.05). A large increase in risk (OR 10.83, 95% CI 2.39–49.2) was observed in women homozygous for haplotype 2 and having a positive family history of breast cancer (Table 5). Similar, although not statistically significant, interactions were seen for age of diagnosis (less than 50 or more than 50, interaction *P *= 0.36) and menopausal status at diagnosis (pre-menopausal or post-menopausal, interaction *P *= 0.19, data not shown). Additional studies focusing on breast cancer incidence in younger, pre-menopausal women would be of interest, to improve the definition of risk associated with this haplotype.

Little is known about the actual effects on the expression or function of these polymorphisms in *BRCA1*. Because of the low complexity of the gene at the haplotype level, we can describe haplotype 2 by using just one SNP, at base pair 64,646. This is in the intron between exons 19 and 20, in the middle of an Alu repeat sequence. This is a rather long intron, spanning 6 kilobases (63,044–69,242). The Alu repeat surrounding base pair 64,646 is a member of the AluSp family. Aligning this sequence against the Alu database at NCBI shows that the consensus nucleotide for this family at this position is G, which is the risk allele. Alignment with other AluSp repeats on the same contig as *BRCA1 *shows that those most similar to this region also have a G at this position. This implies that the G allele might recombine more readily than the A allele with other Alu repeats in this region. It could therefore be hypothesized that this SNP is influential in Alu-mediated non-homologous recombination and other rearrangements of the *BRCA1 *gene. These sorts of aberrations are responsible for roughly 10% of *BRCA1 *disease-causing mutations, and could be involved in somatic alteration of the structure of the *BRCA1 *gene [11]. However, it is important to note that this SNP was selected not because of any prior knowledge of potential function but because it tags a common haplotype.

This risk haplotype is a subset of the wild-type haplotype, and no coding or potential splice-site SNPs in the NIEHS Environmental Genome Project database are in LD with the SNP defining this haplotype. It should be noted that the sequencing data reported by the NIEHS Environmental Genome Project are limited to about 1 kilobase of sequence 5' to the start of transcription, and although the entire 3' untranslated region has been sequenced, only about 800 base pairs beyond the poly(A) site are included. Additionally, 13,403 of 82,899 base pairs (16%) of the genomic region of *BRCA1 *was not sequenced. However, all the unsequenced regions are intronic. This leaves the possibility that a potentially functional SNP in the unsequenced regions of the *BRCA1 *gene resides on haplotype 2.

Osorio and colleagues [12] examined the occurrence of mutations in BRCA1 among the index cases of familial breast and ovarian cancers. They found that mutations occur more readily on the rarer of the two common haplotypes of BRCA1 (their haplotype II). These haplotypes are the third, fourth and fifth listed in Table 3, not the haplotype for which we observed an increase in risk, so the relevance of their observation for our findings is unclear.

Although we cannot rule out the possibility that these results are spurious or due to population stratification, the Nurses' Health Study consists almost entirely of Caucasian women; population stratification should therefore be minimal. Two additional hypotheses that need further examination are that there are functional polymorphisms in the *BRCA1 *gene that are not in the coding sequence, and/or that variants in *BRCA1 *are in LD with functional variants in neighboring genes. The LD block around *BRCA1 *is quite extensive [13], and includes a *BRCA1 *pseudogene, as well as the genes *NBR1 *and *NBR2*. Potentially functional variation in these genes also needs to be described.

## Conclusions

We have described a haplotype associated with the *BRCA1 *gene that is associated with an approximately 20% increase in risk of sporadic breast cancer in the general population. However, the functional variant(s) responsible for the association are unclear.

## Abbreviations

CI = confidence interval; htSNPs = haplotype tagging single nucleotide polymorphisms; NIEHS = National Institute of Environmental Health Sciences; LD = linkage disequilibrium; OR = odds ratio.

## Competing interests

The author(s) declare that they have no competing interests.

## Authors' contributions

DGC carried out analyses and wrote the manuscript, SEH provided funding for the Nurses' Health Study blood cohort and participated in manuscript editing, PK provided statistical support and participated in manuscript editing, DJH provided funding for genotyping and participated in manuscript editing. All authors read and approved the final manuscript.

## Figures and Tables

**Table 1:** Basic characteristics of cases and controls

Characteristic	Cases (*n *= 1322)	Controls (*n *= 1908)
First-degree family history of breast cancer (%)	256 (19)	242 (13)
History of benign breast disease (%)	853 (65)	972 (51)
Post-menopausal status (%)	1133 (91)	1691 (93)
Ever used post-menopausal hormone (%)	902 (76)	1195 (68)
Age at diagnosis (selection in controls, SD)	62.9 (7.4)	63.4 (7.1)
Age at menopause (SD)	48.2 (5.8)	47.8 (6.2)

**Table 2:** Relation of Q356R and breast cancer risk in the Nurses' Health Study

Genotype	Case (frequency)^a^	Control (frequency)^a^	OR (95% CI)	OR^b ^(95% CI)
Q356Q	1065 (86.23)	1413 (87.01)	1.00 (reference)	1.00 (reference)
Q356R	165 (13.36)	206 (12.68)	1.06 (0.85–1.32)	1.05 (0.84–1.32)
R356R	5 (0.4)	5 (0.31)	1.33 (0.38–4.59)	1.67 (0.44–6.35)

CI, confidence interval; OR, odds ratio.

^a^Samples lacking genotype information were removed from analysis.

^b^Logistic regression controlling for age, age of menopause, post-menopausal hormone use, age at first birth, parity, family history of breast cancer, and history of benign breast disease.

**Table 3:** Relation of common *BRCA1 *haplotypes to risk of breast cancer in the Nurses' Health Study

Haplotype^a^	Case^b ^(frequency)	Control^b ^(frequency)	OR (95% CI)	OR^c ^(95% CI)
C A G A	1195(46)	1637 (48)	1.0 (reference)	1.0 (reference)
C A G G	536 (20)	623 (18)	1.19 (1.03–1.37)	1.18 (1.02–1.37)
T A A A	233 (9)	281 (8)	1.11 (0.95–1.29)	1.13 (0.96–1.32)
T A G A	273 (10)	403 (12)	0.92 (0.77–1.10)	0.94 (0.78–1.13)
T G G A	384 (15)	475 (14)	1.15 (0.95–1.39)	1.13 (0.93–1.37)

CI, confidence interval; OR, odds ratio.

^a^Order of SNPs: 33420 (rs799917, P871L), 38085 (rs8176166), 44059 (rs3737559), 64646 (rs8176267).

^b^Samples lacking genotype information at all four SNPs were removed from haplotype analysis.

^c^Logistic regression controlling for age, age of menopause, post-menopausal hormone use, age at first birth, parity, family history of breast cancer, and history of benign breast disease. ORs represent risk increase per copy of each haplotype carried. *P *for global test = 0.08.

**Table 4:** Relative risk of breast cancer by haplotype 2 status in the Nurses' Health Study

Diplotype	Case (frequency)	Control (frequency)^a^	OR (95% CI)	OR^b ^(95% CI)
Other/other	832 (63)	1137 (66)	1.00 (reference)	1.00 (reference)
Hap2/other	429 (33)	531 (31)	1.11 (0.95–1.30)	1.09 (0.92–1.28)
Hap2/Hap2	53 (4.0)	47 (2.7)	1.62 (1.07–2.49)	1.62 (1.05–2.48)

CI, confidence interval; Hap2, haplotype 2; OR, odds ratio.

^a^Samples lacking genotype information at all four SNPs were removed from haplotype analysis.

^b^Logistic regression controlling for age, age of menopause, post-menopausal hormone use, age at first birth, parity, family history of breast cancer, and history of benign breast disease. *P *value for trend = 0.05.

**Table 5:** Haplotype 2 and risk of breast cancer by family history of breast cancer in the Nurses' Health Study

Family history	Case (frequency)^a^	Control (frequency)^a^	OR (95% CI)	OR^b ^(95% CI)
None				
Other/other	668 (51)	995 (58)	1.00 (reference)	1.00 (reference)
Hap2/other	351 (27)	456 (27)	1.16 (0.97–1.38)	1.13 (0.95–1.35)
Hap2/Hap2	39 (2.9)	43(2.5)	1.36 (0.86–2.15)	1.34 (0.84–2.15)
Present				
Other/other	165 (12)	145 (8)	1.69 (1.33–2.17)	1.78 (1.38–2.29)
Hap2/other	77 (5.8)	75 (4.4)	1.51 (1.07–2.12)	1.60 (1.13–2.27)
Hap2/Hap2	14 (1.1)	3 (0.2)	10.06 (2.25–45.0)	10.83 (2.39–49.2)

CI, confidence interval; Hap2, haplotype 2; OR, odds ratio.

^a^Samples lacking genotype information at all four SNPs were removed from haplotype analysis.

^b^Logistic regression controlling for age, age of menopause, post-menopausal hormone use, age at first birth, parity, and history of benign breast disease. *P *interaction = 0.05.
